# Glucocorticoid-Mediated Enhancement of Glutamatergic Transmission May Outweigh Anti-Inflammatory Effects under Conditions of Neuropathic Pain

**DOI:** 10.1371/journal.pone.0091393

**Published:** 2014-03-11

**Authors:** Glenn-Marie Le Coz, Fernand Anton, Ulrike Hanesch

**Affiliations:** Laboratory of Neurophysiology & Psychobiology, University of Luxembourg, Luxembourg, Luxembourg; University of Würzburg, Germany

## Abstract

At the clinical level comorbidity between chronic pain and dysfunctional hypothalamus-pituitary-adrenal (HPA) axis is well established. We aimed to identify causal relationships in a model of neuropathic pain (chronic constriction injury, CCI) by studying the effects of glucocorticoid receptor agonist (dexamethasone) and antagonist (RU-486) administration on pain behavior and spinal biochemical mediators. Daily injections were performed in Sprague Dawley rats. Weight, plasma corticosterone levels and mechanical pain thresholds were assessed before and during 21 days post-CCI. At days four and 21 we investigated the mRNA expression of spinal mediators. In the dexamethasone-injected group, we observed a diminution of body weight and plasma corticosterone levels during the 21 days post surgery period and a more pronounced pain sensitivity until day 7 post-CCI. This enhanced pain sensitivity in the early period following nerve injury was accompanied by a transient increase of the glutamate receptors mGluR5 and NMDA at day 4. However, at this time point we did not observe any effect of the agonist/antagonist injections on the mRNA expression of pro-inflammatory cytokines. The RU-486-injected rats showed a slight mechanical hypoalgesia until 7 days post-CCI, but without any significant correlation with the expression of the measured markers. Our results indicate that glucocorticoid-related modulations of neuropathic pain processing may rather depend on a modification of glutamatergic transmission than on a change in pro-inflammatory cytokine expression.

## Introduction

Comorbidities between chronic pain states and a dysfunctional hypothalamus-pituitary-adrenal (HPA) axis have repeatedly been described [1], [2]. In this framework, research has mainly focused on relationships between adrenocortical reactivity and inflammatory pain states [3], [4]. Recently, experimental studies have used pharmacological approaches to confirm that altered glucocorticoid processing may have a causal influence on pain sensitivity [5], [6]. A reduced adrenocortical reactivity (relative hypocortisolism) that may e.g. be related to chronic exposure to stress may hence lead to an exacerbation of pain [7].

Under physiological conditions, the activation of the HPA axis leads to the production of glucocorticoids (GCs), steroid hormones that bind to two kinds of receptors, the mineralocorticoid receptors (MR) and the glucocorticoid receptors (GR) [8]. The GCs can act at the transcriptional level *via* the translocation of the complex GC-GR to the nucleus which will in turn lead to an enhancement or repression of the transcription of specific target genes [9], [10]. The GCs are known to have an impact on neuropathic pain, especially through their GR receptor, which is upregulated after chronic constriction injury (CCI) [11], [12]. Allodynia and hyperalgesia were prevented by a treatment with a GR antagonist, RU-486 while a GR agonist (dexamethasone) administration exacerbated the pain sensitivity in CCI rats [11].

In neuropathic pain models, an inflammatory component is involved, especially in CCI [13], [14]. Potential effects of pro-inflammatory cytokines should hence be taken into account. Due to the activation of glial cells during central and peripheral sensitization, a release of pro-inflammatory cytokines having a major impact on neuronal activation, on glial cell activity and on the secretion of other factors from these cells has been observed. TNFα is known to be implicated in the initiation and maintenance of central sensitization [15] and to enhance neuronal sensitivity to thermal and mechanical stimulation [16]. Il-1β is well known to increase calcium conductance to the intracellular milieu through the NMDA receptor in nociceptive spinal cord neurons [17]. The GCs have powerful anti-inflammatory effects that could e.g. reduce the pain response *via* an inhibition of the release of the mentioned pro-inflammatory cytokines [18].

Additional biochemical pathways depending on glucocorticoids and significantly altering nociceptive processing have mainly been described in neuropathic pain models. The activation of GR can impact on the glutamatergic system through an upregulation of NMDA receptors and a downregulation of the excitatory amino-acid transporter EAAT3 [11], [19], [20]. In contrast to the inhibition of cytokine release and concomitant reduction of pain sensitivity, GR activation may hence also lead to an enhancement of glutamatergic transmission resulting in exacerbated nociceptive processing. In this case, the glucocorticoids play an important active role in central sensitization, participating in the initiation of neuropathic pain and facilitating the accumulation of extracellular glutamate [21]. In a normal configuration, glutamate is removed from the synaptic cleft by transporters located on neurons (EAAT3) and on glial cells (EAAT2). Besides the involvement of the ionotropic NMDA receptor, the metabotropic receptor mGluR5 has also been demonstrated to be implicated in the central sensitization induced by a CCI model of neuropathic pain [22].

The goal of the present pharmacological study was to investigate the impact of GC on spinal markers of nociceptive processing in a model of neuropathic pain. To mimic states of hyper- and hypo-cortisolism, we used daily administrations of GR agonists (dexamethasone) and antagonists (RU-486) in rats with chronic constriction injury.

We measured metabolic parameters, such as the weight and the plasma corticosterone levels, as well as the repercussion of these treatments on mechanical pain thresholds. Concerning potentially involved biochemical cascades, we investigated markers of the two opposite pathways described above. More specifically, we studied the mRNA expression of the pro-inflammatory cytokines TNFα and IL-1β, of ionotropic (NMDA, through its subunits NR1 and NR2a) and metabotropic (mGluR5) glutamate receptors as well as of the glial and neuronal glutamate transporters EAAT2 and EAAT3.

## Materials and Methods

### Animals

Experiments were performed in 50 adult 50–60 days old male Sprague Dawley rats weighing 207–382 g. Male rats were used to avoid effects of estrogens on glucocorticoid release. The animals (Harlan Laboratories, Netherlands) were housed three per cage in a temperature-controlled room (20–22°C) under a 12 h day-night cycle. Food and water were provided *ad libitum*. The body weight was regularly controlled. Starting two weeks before initiation of the experiments, the animals were handled daily and habituated to the behavioral testing room and devices. The Animal Care and Use Committee of the University of Luxembourg approved all animal procedures.

### CCI surgery

For chronic constriction injury (CCI) surgery, all rats were anesthetized with isoflurane using an anesthesia unit (Univentor 400, Zejtun, Malta). The right sciatic nerve was exposed and three natural chromic gut 4–0 (Stoelting Europe, Dublin, Ireland) loose ligatures were placed around the nerve. The distance between two ligatures was 1 mm. The muscle layer was closed with 4–0 silk sutures and the skin layer was closed with surgical skin staples.

### Experimental protocol

The rats were divided into three groups receiving daily injections of either dexamethasone (DEX, n = 16), or RU-486 (RU, n = 17) or vehicle (CON, n = 17) from two days before the surgery until the end of the experiment. Ten animals per group were sacrificed already 4 days after the CCI surgery to measure early changes in the expression level of spinal mediators. The remaining rats (DEX: n = 6, RU: n = 7, CON: n = 7) underwent a long term experiment. They were sacrificed at day 21 post surgery and their spinal cord was also examined for spinal mediator mRNA.

The course of the experiments was as follows: mechanical pain thresholds were assessed on three consecutive days (d-5, d-4 and d-3) to reveal baseline values (BS). The daily pharmacological treatment started at d-2 and on this day and the two following ones (d-2, d-1, d0) mechanical thresholds were also measured (baseline with injections, BSi) to test for drug effects. At d0 CCI surgery was performed and pain sensitivity measurements were done on days 4, and for the long term groups additional on days 7, 10, 14, and 21. Blood sampling was carried out on d-2 (BS), d0 (BSi) and on days 1, 4, and 7, 14 and 21 following CCI surgery. Animals were sacrificed after the experiments on day 4 or 21 and the spinal cord was removed and processed for qPCR. Weight, corticosterone levels and behavioral data of the animals sacrificed at day 4 were in the same range as those measured in the long term group. For this reason only the data covering the 21 days of experiment are presented.

### Von Frey monofilament test

The Von Frey test was used to measure mechanical allodynia/hyperalgesia. Rats were placed in Plexiglas® cages with wire mesh bottoms and given 10 min to acclimate. Each monofilament (OptiHair, MarstockNervTest, Germany) was placed perpendicularly onto the midplantar region of the hind paw and pressure was increased until the point of deflection of the filament was reached. Pain thresholds were determined by the ascending and descending method of limits with forces ranging from 8 to 256 mN in 11 logarithmic steps [23]. Each test was repeated three times and the mean was calculated for each paw.

We set the values of the left unaffected side (control) to 100% and expressed the threshold of the right injured paw as a percentage of the left one.

### Blood sampling and measurement of corticosterone levels

Blood sampling was performed in awake animals between 9 h and 9 h30, half an hour before the injections. To minimize stress reactions, rats were loosely held in a towel and then a small incision at about 15 mm from the end of the tail was performed using a scalpel. 300 µl of blood was collected with a capillary tube coated with EDTA (Microvette CB300, Sarstedt, Essen, Belgium). The samples were immediately placed on ice, then centrifuged for 10 min at 4°C and the plasma was removed and stored at −20°C.

Serum corticosterone levels were measured using an ELISA (Enzyme-Linked Immunosorbent Assay) kit (Assay Designs, USA). Analysis was done according to the manufacturer's protocol. Data were acquired via a plate reader (Sunrise Magellan, Austria) using an adequate software package (Magellan software, v6.4 standard, Austria).

### Injections of dexamethasone, RU-486, or vehicle

The first day of injection (d-2) preceded the surgery by two days and the injections were repeated daily until the end of experimentation at d4 or d21. The groups received daily intraperitoneal injections of either dexamethasone, a GR agonist (dexamethasone 21-phosphate disodium salt, Sigma Aldrich, Germany), at a dose of 0.5 mg/kg/d dissolved in sterile saline 0.9%, or RU-486, a GR antagonist (mifepristone, Sigma Aldrich, Germany), at a dose of 4 mg/kg/d liquefied in olive oil containing 1% ethanol. The control group was treated either with olive oil containing 1% ethanol (8 animals) or saline 0.9% (9 animals). Since these two control groups did not differ in their results, we pooled the data. The daily injected volume was 0.2 ml. Injections were always performed at the same hour (10 am), 30 min before starting the behavioral tests.

### Sample and RNA collection

At day 4 or 21 post surgery, rats were deeply anesthetized with isoflurane and decapitated. Levels L4/L5 of the spinal cord were removed and the total RNA was extracted with the Invitrap Spin Tissue RNA Microkit (Invitek, Germany). The RNA concentration was determined by measuring the absorbance at 260 nm, using a Nanodrop ND-2000 spectrophotometer (Thermo Fisher Scientific, Wilmington, U.S.A.).

### Reverse transcription and Real-Time qPCR

Total RNA (500 ng) was reverse transcribed into cDNA using the ImProm-II Reverse Transcription System (Promega Corporation, Madison, USA). Real-Time PCR reactions were performed from 10 ng of cDNA with a CFX-96 thermocycler (Bio-Rad Laboratories, Nazareth, Belgium) using SYBR green Supermix PerfeCTa (95053–02K, Quanta Biosciences, Gaithersburg, MD, USA) and primers for the reference gene, β-actin, and the genes of interest presented in Table 1.

**Table 1:** Sequences of primers used in this study.

Name	Accession	Sequence
β-actin	NM_031144	5′ GCT GAG AGG GAA ATC GTG CGT GAC 3′
		5′ GGA GGA AGA GGA TGC GGC AGT GG 3′
EAAT2	NM_017215	5′ ATG CTC CTC ATT CTC ACA G 3′
	NM_001035233	5′ CTA CAT TGA CCG AAG TTC TC 3′
EAAT3	NM_013032.3	5′ TCA TAG TCG GGA AGA AC 3′
		5′ AGC GGA ATG TAA CTG GAA GG 3′
IL-1β	NM_031512	5′ GTT GAA TCT ATA CCT GTC CTG TG 3′
		5′ TGG TCT TGA CTT CTA TCT TGT TG 3′
TNFα	NM_012675	5′ GCT CTT CTG TCT ACT GAA CTT C 3′
		5′ GAT CTG AGT GTG AGG GTC TG 3′
mGluR5	NM_017012.1	5′ ACA ACC TCT ACA GTG GTA CG 3′
		5′ GGC CCA AGT CAC AGA TTT TC 3′
NR1	NM_001270607	5′ GGT TGC GTG GGC AAC ACC AA 3′
		5′ CCG TCC GCA TAC TTA GAA GA 3′
NR2a	NM_012573.3	5′ CAG ATA ACA ATA AGA ACC ACA AG 3′
		5′ AAC ATC GCT ACA GTC CTT 3′

The steps consisted of one cycle of 3 min at 95°C and 40 cycles of amplification (10 sec at 95°C, 30 sec at 61°C). All samples were run in triplicate. Relative expression was estimated using the ΔΔCt- method [24] with β-actin as the reference. Threshold cycle values (CT) were used to compute the amount of target gene mRNA in relation to the reference gene mRNA (β-actin). ΔCT represents the difference between the number of cycles that were necessary to detect the PCR products of the target and the reference genes. The ΔΔCT indicates the difference between the ΔCT of the pharmacologically treated groups and the ΔCT of the vehicle treated (CON) animals. The data were expressed as 2^−ΔΔCT^ and the mean of the right injured as well as of the left not affected side was computed for each group. Since we did not notice any significant differences in the relative expression levels, we only present the data for the right affected side of the spinal cord.

### Statistical analysis

Statistical analyses for mechanical thresholds and qPCR were carried out using an analysis of variance (ANOVA) followed by a Scheffé *post hoc* test to identify differences between groups. Regarding the weight and corticosterone levels a Dunnett T3 *post hoc* test was used. P<0.05 was defined as the level of statistical significance. Statistical tests were performed with IBM SPSS Statistics version 19 (IBM corporation, Somers, NY, USA).

## Results

### Effect of GR-antagonist/agonist administration and CCI surgery on metabolism

**Figure 1:**
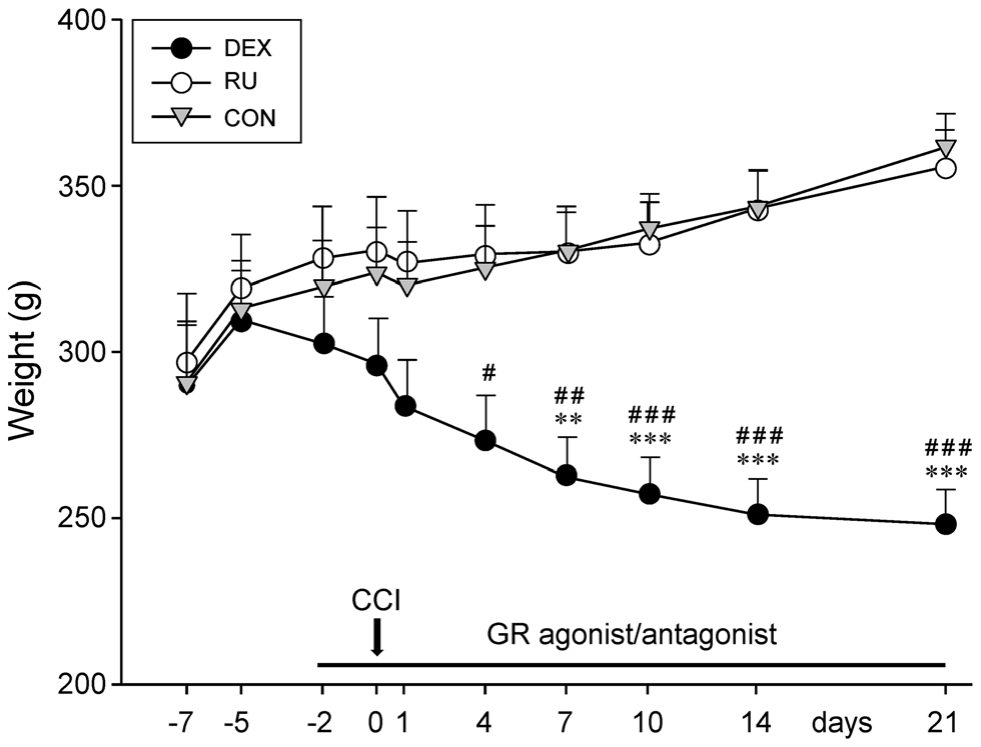
Animal weight changes following GR-agonist/antagonist treatment and CCI. Influence of daily injections of the GR agonist dexamethasone (DEX), the GR antagonist RU-486 (RU), or vehicle (CON) in combination with chronic constriction injury (CCI) on the rat weight. Administration of the GR agonist resulted in a constant loss of weight, unaffected by CCI surgery. GR antagonist or vehicle treated animals exhibited a normal gain of weight, only slightly slowed down by the CCI surgery. Data are shown as mean ± s.e.m. **p<0.01, ***p<0.001 indicate significant differences between DEX and RU and ^#^p<0.05, ^##^p<0.01, ^###^p<0.001 represent significant differences between DEX and CON.

The weight of the rats was followed up from 7 days before to 21 days post-CCI at different time intervals (Fig. 1). At the start of the experiment, at d-7, the mean weight was identical in all three groups (DEX: 289.83±18.25 g, RU: 297.33±20.18 g, CON: 291±18.15 g). A normal increase in weight was then observed at d-5 (DEX: 309.75±14.60 g, RU: 319.17±16.20 g, CON: 313.17±14.24 g).

With the start of the drug injections the animal weight in the DEX group decreased constantly, regardless of the CCI surgery, until the end of the experiment at d21 (248.27±10.33 g). In contrast, the rats in the RU group gained weight until d21 (355.75±11.05 g) in a time course comparable to the one seen for CON (d21: 361.83±10.03 g). In the two latter groups the nerve constriction on d0 only slightly slowed down the increase in weight.

Statistical analysis revealed a significant difference between the mean weight of DEX animals and CON from d4 (p<0.05) to d21 (p<0.001) and between DEX and RU rats from d7 (p<0.01) until the end of the experiment at d21 (p<0.001).

### Alterations of plasma corticosterone concentrations in response to GR-antagonist/agonist injections and CCI surgery

**Figure 2:**
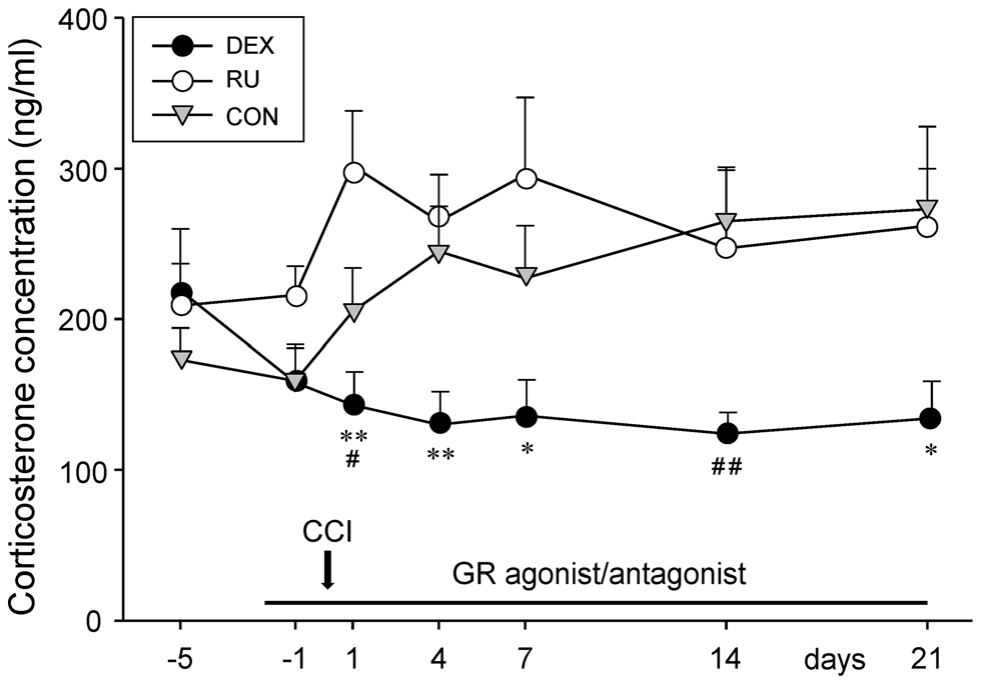
Changes in blood corticosterone concentration throughout drug administration and CCI periods. Despite CCI surgery, daily administration of the GR agonist dexamethasone (DEX) led to a slight decrease of plasma corticosterone levels, persisting throughout the experiment. Injections of the GR antagonist RU-486 (RU) or vehicle (CON) *per se* had no effect on the concentration of circulating corticosterone. The chronic constriction injury (CCI) however triggered a persistent increase in plasma corticosterone levels. Data are presented as mean ± s.e.m. *p<0.05, **p<0.01 indicate significant differences between DEX and RU and ^#^p<0.05, ^##^p<0.01 between DEX and CON.

The three groups of rats displayed comparable plasma corticosterone levels at d-5 before the administration of the GR-agonist or -antagonist (DEX: 219±41 ng/ml, RU: 209±28 ng/ml, CON: 173±21 ng/ml) (Fig. 2).

Injections of vehicle (CON group) did not alter the blood corticosterone levels *per se* (d-1: 159±22 ng/ml). The nerve injury on d0 however resulted in an increase of the corticosterone concentration from d1 (205±29 ng/ml) to the end of the experiment (d21: 273±55 ng/ml). In the DEX group the concentration of circulating corticosterone decreased following the administration of dexamethasone and persisted at this low level throughout the course of the experiment despite the CCI surgery. Compared to the CON animals the DEX group exhibited significantly lower corticosterone levels at d1 (p<0.05) and d14 (p<0.01) but not at d7 and d21.

In contrast, injections of the GR-antagonist RU-486 did not lead to alterations in blood corticosterone levels in the pre-surgery period (d-1: 216±19 ng/ml). However, the nerve constriction on d0 led to an increase in the concentration of circulating corticosterone measured on d1 (302±36 ng/ml). Throughout the course of neuropathy the levels remained enhanced. In comparison with the control group no significant differences were seen.

Comparing the DEX and RU groups statistically significant differences were observed at all time points after CCI surgery except at d14 (d1: p<0.01; d4: p<0.01; d7: p<0.05; d14: p = 0.129; d21: p<0.05), confirming a strong effect of dexamethasone on the plasma corticosterone levels.

### Effect of systemic GR-antagonist/agonist treatment on nerve injury-related mechanical allodynia/hyperalgesia

**Figure 3:**
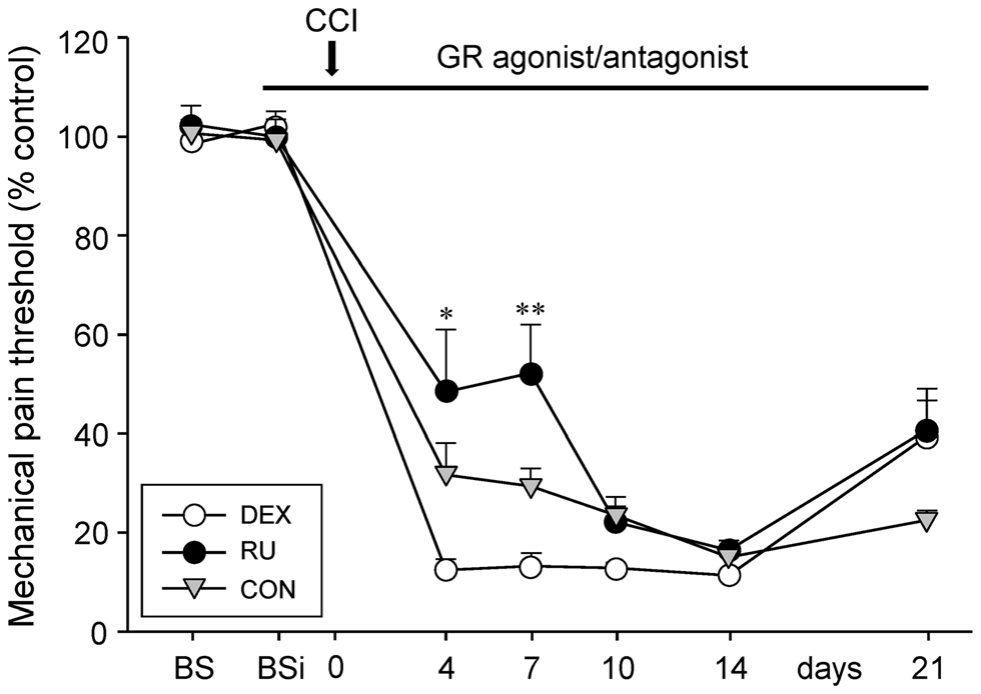
Influence of GR-agonist/antagonist treatment and CCI on mechanical pain thresholds. Treatment of the animals with either GR agonist dexamethasone (DEX), GR antagonist RU-486 (RU), or vehicle (CON) did not change the mechanical sensitivity *per se*. Chronic constriction injury (CCI) resulted in an increase of pain sensitivity in all three groups. Dexamethasone receiving rats were more sensitive to mechanical stimulation than RU-486 injected rats up to seven days post surgery. Data of the right affected paw are presented as percentage of the left control paw (mean ± s.e.m.). Asterisks indicate a significant difference between the DEX and RU group for the individual time point (* p<0.05, ** p<0.01).

On the three consecutive days (d-5 to d-3) preceding any manipulation, rats were tested for baseline mechanical paw withdrawal (BS). The pain thresholds were expressed in percentages of the right injured paw compared to the left paw that served as control (Fig. 3). As expected, no difference between the three groups could be observed (DEX: 98.5±4.92%, RU: 102.5±3.79%, CON: 100.7±1.66%). Thereafter, animals were injected daily and pain thresholds were again assessed during the three following days (d-2 to d0) to obtain a baseline under conditions of drug application (BSi  =  baseline with injections). We did not observe any significant differences between the groups neither under BSi conditions (DEX: 102.6±2.57%, RU: 100±1.26%, CON: 99.3±4.24%), nor under BS control conditions.

Four days after CCI we noticed, as expected, a pronounced nerve injury-related increase in pain sensitivity for all three groups. However, the degree of increase differed between groups. Whereas the RU animals appeared less sensitive (48.4±12.59%) to noxious mechanical stimulation than CON (31.7±6.43%), the DEX rats exhibited a higher pain sensitivity (12.4±2.04%). This differential pain sensitivity of the groups was still seen 7 days after surgery (RU: 52.20±9.80%, CON: 29.4±3.58%, DEX: 13.2±2.64%). At d10 and 14, the pain thresholds of RU (16.4±1.95%) and CON (15±1.13%) further decreased, reaching the same level as DEX (11.3±0.46%). Only at d21, the mechanical pain sensitivity slightly decreased in all three groups (DEX: 39.5±9.54%, RU: 40.8±5.83%, CON: 22.5±1.75%).

Intergroup comparisons revealed that rats receiving systemic RU 486 treatment were less sensitive to noxious mechanical stimulation for up to 7 days after CCI surgery than rats with dexamethasone administration (d4: p<0.05; d7: p<0.01).

### Alteration of cytokine expression following dexamethasone or RU-486 treatment

**Figure 4:**
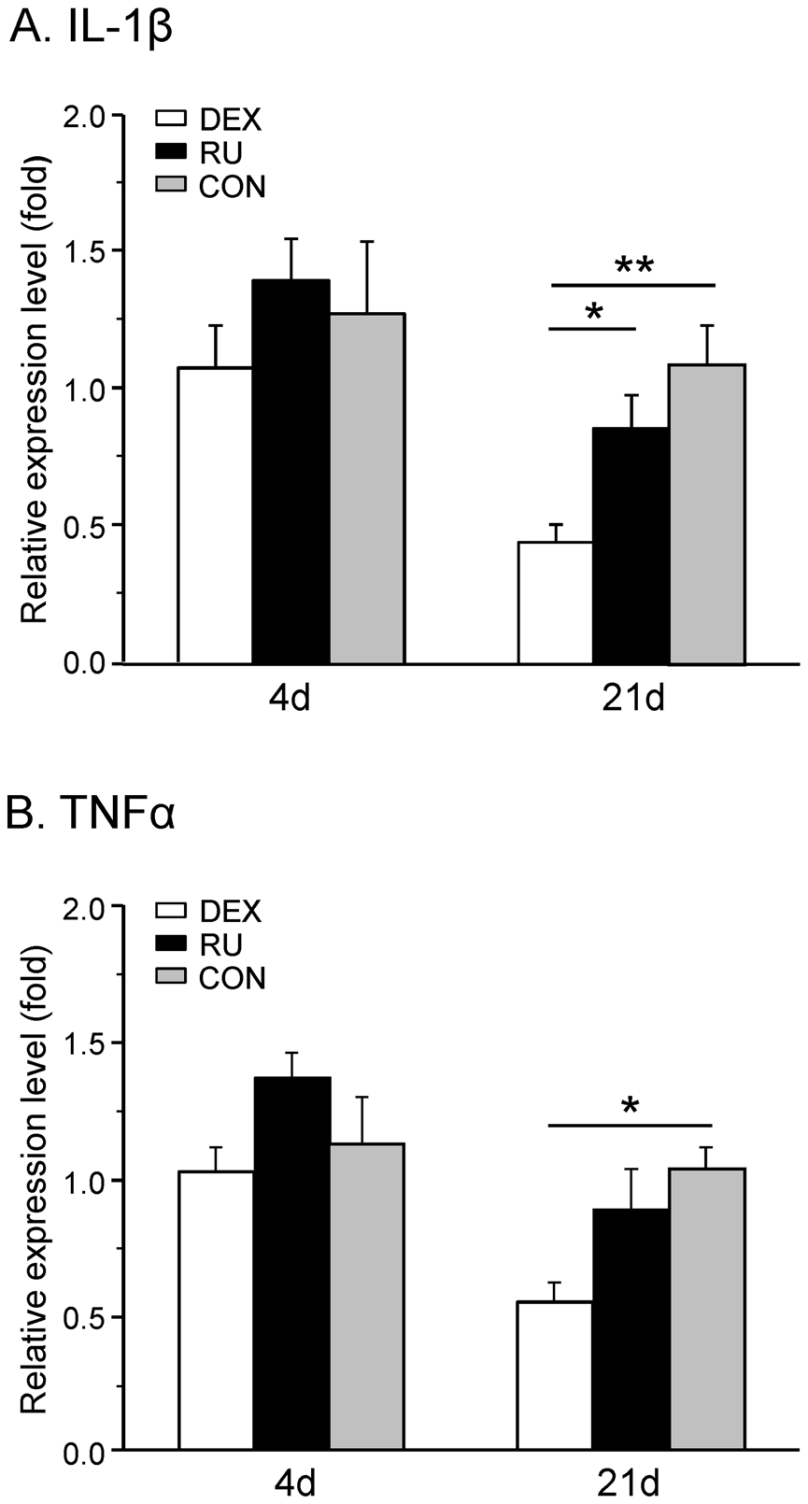
Spinal cytokine mRNA expression following CCI in dexamethasone and RU-486 treated rats. The relative mRNA expression levels of (**A**) IL-1β and (**B**) TNFα in the right spinal cord segment L4/L5 were measured at days four and 21 after CCI surgery in three groups of rats receiving either daily dexamethasone (DEX), RU-486 (RU), or vehicle (CON) injections. Early after injury activation or inhibition of GR did not alter the relative expression levels of both cytokines. In the later phase, the mRNA of IL-1β was downregulated by dexamethasone and RU-486 treatment, whereas TNFα mRNA levels were reduced only by GR activation. Data are expressed as mean ± s.e.m. *p<0.05, **p<0.01, ***p<0.001.

IL-1β and TNFα are cytokines known to be implicated in neuropathic pain mechanisms. Four days after surgery the IL-1β mRNA expression did not display any difference in the three groups (DEX: 1.36±0.21, RU: 1.17±0.14 and CON: 1.32±0.35) (Fig. 4A). At d21 post-CCI the expression of this cytokine was reduced in the DEX group (0.41±0.06) but not significantly changed in rats treated with RU-486 (0.79±0.11) in comparison with the control group (1.00±0.13). Statistical analysis revealed a significant difference at day 21 between DEX and CON (p<0.01) and also between DEX and RU (p<0.05).

We could observe the same temporal regulation profile for TNFα. At d4, no significant up- or downregulation of the mRNA expression of this cytokine was observed in the treated groups (DEX: 0.83±0.10, RU: 1.53±0.19 and CON: 1.05±0.11). 21 days post surgery the mRNA expression was downregulated in the DEX group (0.54±0.07) but not in the RU rats (0.87±0.15) (Fig. 4B). At this time point we observed a significant difference between the DEX group and CON (p<0.05).

These data confirm a (late) regulatory effect of dexamethasone but not of RU-486 on the mRNA expression of the two studied pro-inflammatory cytokines.

### Impact of GR-agonist/antagonist administration on components of the glutamatergic system

#### Expression of excitatory amino-acid transporters

Excitatory amino-acid transporters (EAATs) also known as glutamate transporters are essential to homeostasis, permitting the uptake of the neurotransmitter from the synaptic cleft to regulate its effects. In the present study, we focused on two of the five subtypes, one, which is located in the membrane of glial cells (EAAT2) and the other which can be found in the membrane of neurons (EAAT3, also known as EAAC1).

**Figure 5:**
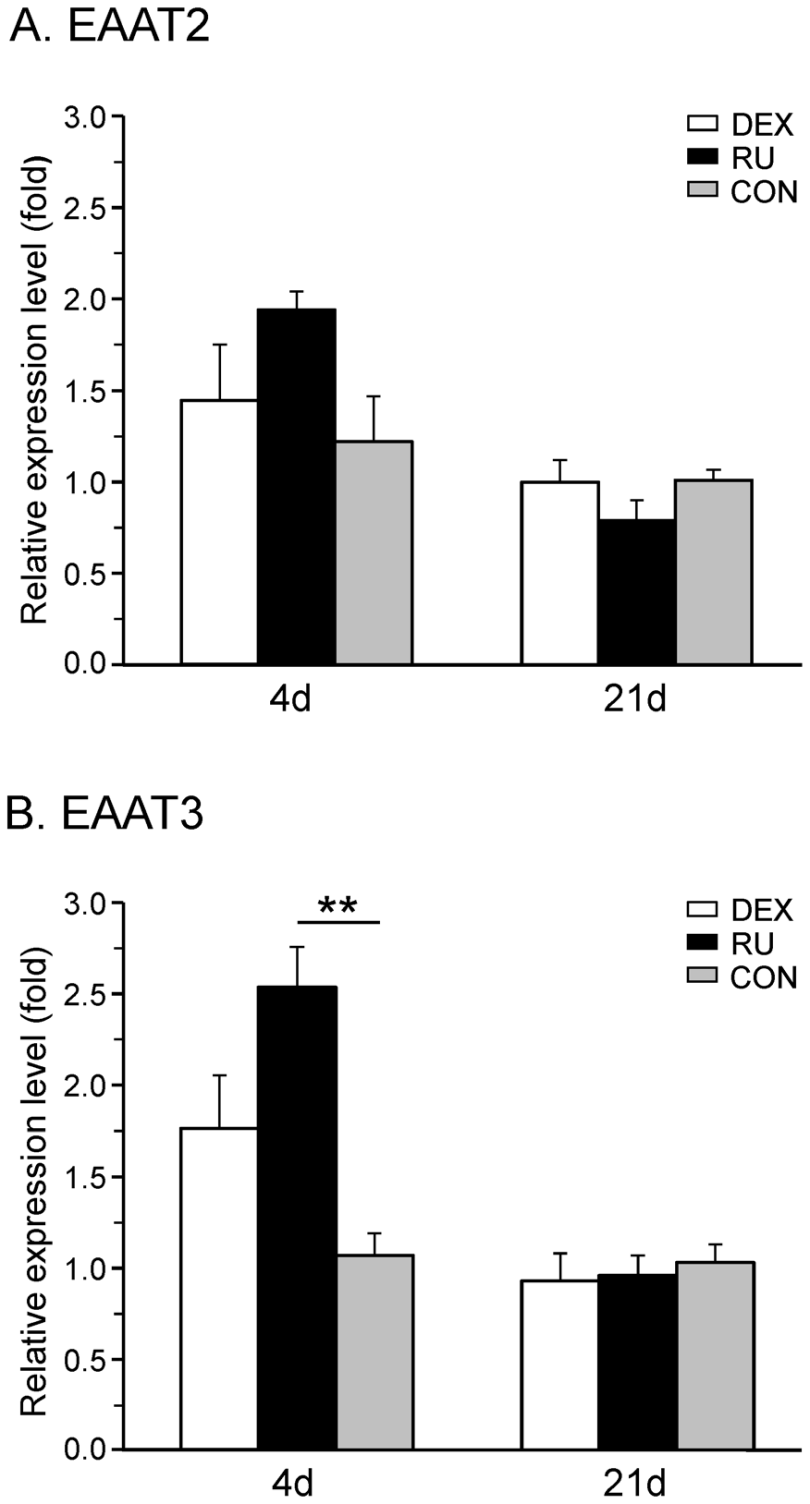
CCI-mediated mRNA expression of spinal EAATs under GR-agonist/antagonist treatment. The relative mRNA expression levels of (**A**) the glial glutamate transporter EAAT2 and (**B**) the neuronal glutamate transporter EAAT3 were measured in the right spinal cord segment L4/L5 at days four and 21 after CCI surgery in three groups of rats receiving daily injections of either GR agonist dexamethasone (DEX), GR antagonist RU-486 (RU), or vehicle (CON). Neither activation nor inhibition of GR significantly influenced the relative expression levels of EAAT2 at both time points (**A**). EAAT3 mRNA was upregulated in the acute phase of neuropathy by RU-486 administration but not by dexamethasone. At later stages, modulation of GR activity did not result in any effect on EAAT3 transcription (**B**). Data are expressed as mean±s.e.m.

The mRNA expression of the glial transporter EAAT2 showed no significant change in the RU group four days (1.94±0.10) and 21 days (0.79±0.11) after surgery (Fig. 5A). Likewise, the administration of the GR-agonist had no statistic effect on the expression of EAAT2, neither under short term (1.45±0.3) nor under long term (1.00±0.12) neuropathic conditions.

Regarding the neuronal transporter EAAT3, no significant alteration in the mRNA expression was found in the DEX group in the early period (1.76±0.29) as well as three weeks following nerve constriction (0.93±0.15). Systemic treatment with RU-486 induced a significant increase in the expression of EAAT3 after four days (2.53±0.22; p<0.01 compared to CON) and came back to control levels at 21 days (0.96±0.11) (Fig. 5B).

Taken together, the systemic administration of the GR-antagonist resulted in a significant increase in mRNA levels of merely the neuronal glutamate transporter EAAT3. In contrast, dexamethasone displayed no significant influence on the regulation of the excitatory amino-acid transporters, neither in the early development nor in later phases of neuropathy.

#### Expression of glutamate receptors

The metabotropic glutamate receptor mGluR5 as well as the ionotropic NMDA receptor are claimed to be involved in neuropathic pain syndromes. For the latter, seven subunits have been described. Here, we focused on the two commonly expressed NR1 and NR2a subunits.

**Figure 6:**
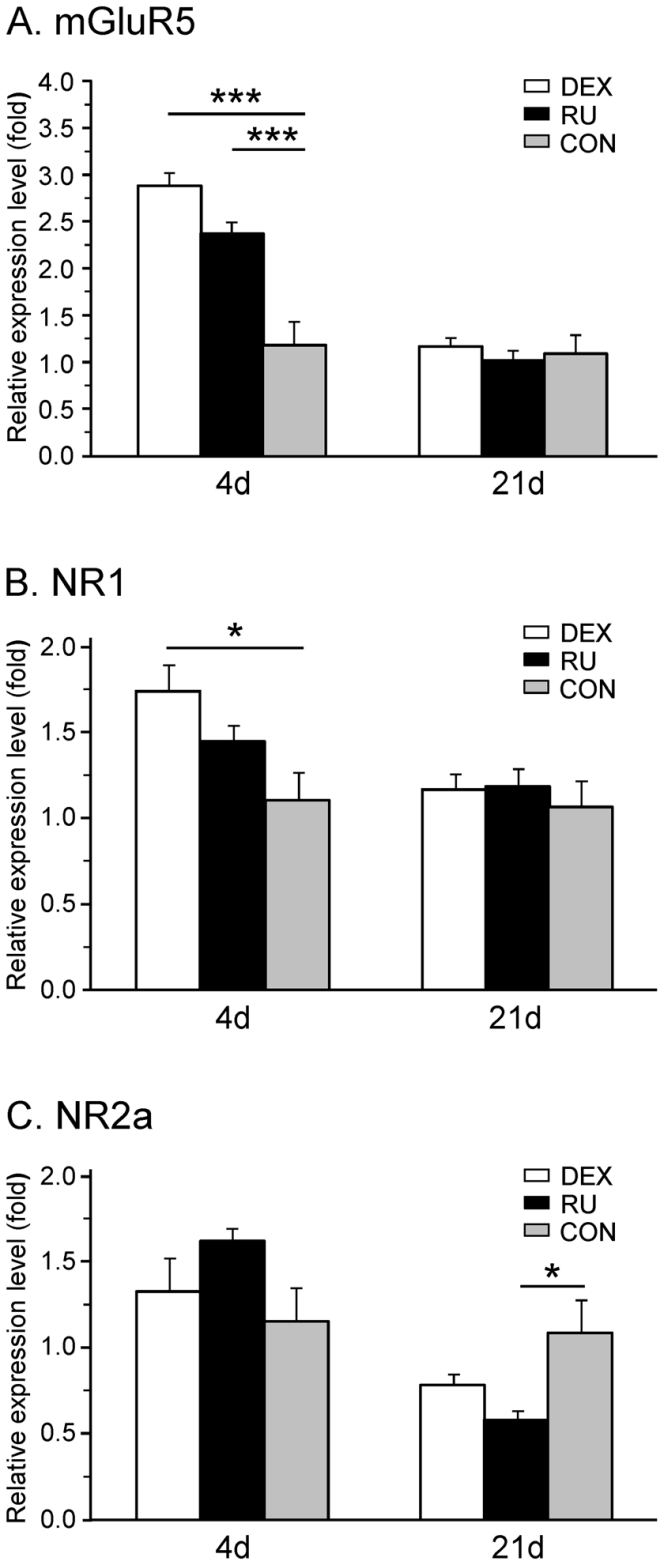
mRNA expression of spinal ionotropic and metabotropic glutamate receptors following GR-agonist/antagonist administration and CCI. The relative mRNA expression levels of (**A**) the metabotropic receptor mGluR5, (**B**) the subunit NR1 and (**C**) the subunit NR2a of the ionotropic NMDA receptor were measured in the right spinal cord segment L4/L5 at days four and 21 after CCI surgery in three groups of rats receiving daily injections of either dexamethasone (DEX), RU-486 (RU), or vehicle (CON). Early after CCI the mGluR5 mRNA was significantly upregulated by GR activation and inhibition, whereas the NR1 subunit was significantly upregulated only in the DEX group. For both receptors no change from control was seen in the late phase of neuropathy. On the other hand, the NR2a mRNA was not altered in acute conditions but downregulated at 21 days in the RU group treated with the GR antagonist. Data are expressed as mean ± s.e.m. *p<0.05.

Regarding the mRNA expression of the metabotropic receptor mGluR5, a transient and significant increase was only seen early after nerve injury in the DEX (2.88±0.14; p<0.001) as well as the RU group (2.37±0.12; p<0.001) as compared to CON (1.18±0.25). At d21 the mRNA expression of mGluR5 came back to control levels in both treatment groups (DEX: 1.17±0.09; RU: 1.02±0.10; CON: 1.09±0.21) (Fig. 6A).

For the NR1 mRNA expression, a significant increase was noticed in the DEX group at d4 (1.73±0.16) compared to CON (1.00±0.16; p<0.05). The level returned to baseline at d21 post surgery (DEX: 1.17±0.09, CON: 1.06±0.15) (Fig. 6B). Administration of the GR-antagonist RU-486 had no significant influence on the NR1 regulation at d4 (1.44±0.09) and d21 (1.02±0.10).

Neither activation nor inhibition of the GR led to any significant modification of the NR2a mRNA expression early after nerve constriction (RU: 1.61±0.07; DEX: 1.32±0.19; CON: 1.15±0.19) (Fig. 6C). However, in the later phase of neuropathy, the RU group displayed a significant decrease of the relative expression level (0.58±0.0; p<0.05) whereas the level measured in the DEX group (0.78±0.06) was not statistically different from control (1.08±0.19).

Summarizing the effects of pharmacological GR-manipulation on the expression of glutamate receptors, we observed an early GR-activation mediated upregulation of metabotropic and ionotropic receptors and a late downregulation of one subunit of the NMDA receptor related to GR-antagonist application.

## Discussion

In the present work, we aimed to determine the role of GCs in the onset and maintenance of mechanical allodynia/hyperalgesia in a CCI model of neuropathic pain. Our observations included: **1**. A decrease in body weight for the DEX group throughout the 21 days following CCI surgery; **2**. A higher sensitivity to painful mechanical stimulation of the DEX group until day 7 post surgery; **3**. A lack of effects of dexamethasone or RU-486 injections on early pro-inflammatory cytokine mRNA expression; **4**. No significant differences in EAAT2 mRNA expression at days 4 and 21 after the CCI surgery but a RU-486-related increase at day 4 for EAAT3; **5**. A pronounced transient increase in NMDA receptor subunit NR1 and in mGluR5 mRNA expression in the DEX group at 4 days post surgery; **6**. A decrease in the NMDA receptor subunit NR2a mRNA expression only 21 days post CCI in the RU group.

### Metabolic effects of GR-agonist or -antagonist administration

In the DEX group consistent decrease in body weight was observed from the starting day of the injections until the end of the experiment. Since glucocorticoids are known to imply the mobilization of fatty acids [8], our findings may be related to dexamethasone mimicking the effects of endogenous GCs and provoking a utilization of the mentioned fatty acids and the concomitant loss of weight. In the present study, plasma corticosterone levels reflecting HPA axis activity under physiological conditions have been modified by the dexamethasone or RU-486 injections. In the first days of the observation, i.e. in the absence of any manipulation, no differences were observed between the three groups. After starting the drug injections, the plasma corticosterone levels of the DEX group decreased and then stayed at a stable level from day one to the end of the experiment. This is consistent with the fact that dexamethasone mimics endogenous GC effects leading to an activation of the negative feedback control of the HPA axis. For the other two groups, the increase observed at day one was probably mainly due to the surgery performed at day zero. At this time point, the dexamethasone administration probably masked these surgery-related variations in the DEX group via the described activation of the negative feedback loop. The corticosterone concentration in the RU and CON groups stayed at a higher and relatively constant level throughout the experiment. This is consistent with data from Soro and colleagues [25] who failed to observe an effect of RU-486 on the corticosterone level of Sprague Dawley rats.

### Development and maintenance of mechanical allodynia/hyperalgesia

The measurement of mechanical allodynia/hyperalgesia during three days without interventions and the three days with drug injections did not reveal any differences between the groups. In the absence of any injury, the administration of GR agonists or antagonists did hence not have any effect on the mechanical pain thresholds. However, 4 to 7 days after the CCI surgery, the RU group was significantly less sensitive than the DEX group. This observation is in accordance with a recent study of Ou and colleagues [26] highlighting a protective effect of another GR antagonist, M8046, in CCI-induced neuropathic pain. The pain thresholds of the CON rats were located between those of the pharmacologically treated groups during the same period. Our observations are consistent with studies showing that intrathecal injections of dexamethasone increase pain-like behaviors in a spared nerve injury (SNI) model [19] and CCI model of neuropathic pain and that GR antagonists like RU-486 attenuate them [20]. These effects are opposite to the well-known anti-inflammatory and hypoalgesic effects of GR activation and may suggest that other pathways involving the glutamatergic system known to have a role in neuropathic pain (for review see [27]) should be more implied in the onset of mechanical allodynia than the local inflammation. No significant differences in pain sensitivity between the groups were detected in the later period following CCI surgery. This could be due to adaptive biochemical processes and to the implication of other mediators coming into play following pronounced ongoing pharmacological activation or inactivation of GRs. It should be noted here that effects related to pharmacological manipulation of the HPA axis have been described to display complex and even paradoxical patterns, depending on factors like dose, site and mode of application and on duration of the treatment [28]. In this context, Kingery and co-workers [29] have e.g. observed that once-daily injections of methylprednisolone had no antihyperalgesic effect in a rat sciatic nerve transection model while continuous infusion reversed heat and mechanical hyperalgesia. More specifically turning to glucocorticoid receptor antagonists, Takasaki and colleagues [12] described that intrathecal and intraperitoneal injections of RU-486 produced antinociceptive effects in a mouse model of neuropathic pain while intracerebroventricular injections had no effect. The authors however only provided short term observations. We are not aware of any data on long term effects in this context. The measurement of the mRNA expression of the mentioned cytokines as well as glutamate transporters and transmitters at d21 was partly aimed at considering the time dependent involvement of implied mediators.

Taken together, our behavioral results seem to emphasize a more important role of GR modulation in the onset of mechanical allodynia/hyperalgesia rather than in the longer term maintenance.

### Involvement of pro-inflammatory cytokines

As mentioned in the final paragraph of the methods section, we did not observe significant side differences in the expression levels of cytokine mRNAs. The same was true for the other examined biochemical markers. This at first astonishing finding is reminiscent of the implication of spinal glia and pro-inflammatory cytokines in mirror-image neuropathic pain [30]. Bilateral labelling of activated microglia in the spinal cord dorsal horn following a unilateral spared nerve injury has e.g. been described in rats [31]. The fact that we did not observe altered pain behavior induced by stimulation of the contralateral (control) paw in spite of the quite symmetrical biochemical expression of mRNA levels may be related to a time dependent dissociation between pain behavior and the measured biochemical parameters. In this sense, Huiging Li and colleagues [32], using a spared nerve injury model in rats, have e.g. observed elevated pro-inflammatory cytokine levels with effects of steroid treatment gradually becoming less apparent while the effects of the same drug on mechanical sensitivity remained evident on day 7 post surgery. In our hands, there may also have been a more pronounced ipsilateral expression of spinal mediators in the initial phase preceding day 4 after CCI which gradually decayed and converged with the respective values of the untreated side.

Pro-inflammatory cytokines such as IL-1β and TNFα play an important role in inflammatory reactions and may also be involved in neuropathic pain [9], [33] and in the initiation and maintenance of central sensitization [34].

We only observed a pronounced decrease in IL-1β mRNA expression at day 21 post CCI surgery for the DEX group, as compared to the two other groups. This is consistent with studies from cell cultures exhibiting an important diminution in IL-1β mRNA expression after corticosteroid administration [9], [35]. Surprisingly, we did not observe any DEX-related effects on the mRNA expression of this cytokine at day 4. This negative finding could reflect that IL-1β may not be involved in the onset of mechanical allodynia following CCI. We did not have the possibility to include measurements of protein levels in the present study. These data could be important for the consideration of potential posttranscriptional regulation-related effects which may play a critical role in the regulation of pro-inflammatory cytokine expression by mitogen activated protein kinase signal transduction pathways as well as by anti-inflammatory agents and mediators (for review see [36]). These may in turn depend on a host of factors that have recently been discussed in detail by Ji and colleagues [37]. The absence of dexamethasone effects at day 4 post CCI is however in line with the enhanced sensitivity to mechanical allodynia/hyperalgesia that may be linked to pro-inflammatory cytokine-mediated effects.

The TNFα mRNA expression was likewise downregulated in the DEX group at day 21. This is also in line with other studies showing a decrease of TNFα expression after administration of dexamethasone [38], [39] and an inhibition of TNFα translation by GCs [40]. The absence of a significant decrease after 4 days is coherent with delayed modifications regarding this pro-inflammatory cytokine. In agreement with our results, an inhibition of spinal TNFα receptor expression following administration of the synthetic GC methylprednisolone has also been shown, an effect that may be reversed by the administration of RU-486 [41].

Taken together, our results are in accordance with the anti-inflammatory effects of GR activation in a later phase. TNFα and IL-1β are known to activate glia cells [42]. Effects of GR activation or inactivation on this type of cells thus remain to be considered in the present context. On the other hand, the fact that the DEX group was more sensitive to mechanical allodynia/hyperalgesia in the early phase after surgery may point to the dominance of GR-mediated enhancement of glutamatergic processing as will be discussed below.

### Role of glutamate transporters

Dexamethasone has been shown to have an inhibitory effect on the downregulation of glutamate transporters such as EAAT1 ( = GLAST), EAAT2 ( = GLT1) and EAAT3 ( = EAAC1) after chronic morphine administration [43]. Along this line Kalariti and colleagues [44] have demonstrated an increase in EAAT1 protein but not mRNA expression *in vitro* following dexamethasone administration. GR-activation has also been reported to diminish EAAT3 expression under conditions of nerve injury, effects reversed by administration of RU 486 [19]. The decreased glutamate transporter expression leads to an increase of extracellular glutamate resulting in amplified synaptic transmission [11], [19], [20].

In the present study we did not observe any significant change of EAAT3 mRNA expression in the dexamethasone treated group at day 4, whereas, at the same time point, the RU group exhibited an important increase. The EAAT2 mRNA expression was not affected, neither by dexamethasone nor by RU-486.

Although pointing towards the same direction, these results are not fully consistent with the studies mentioned above. These discrepancies may be related to the different models used to induce neuropathic pain as well as to the doses of the respective drugs that have been administered. The RU-486 injection-related increase is however in accordance with the literature. It may participate in the enhancement of glutamate uptake from the synaptic cleft leading to a reduction of glutamatergic transmission and of pain sensitivity as observed in the RU group at days 4 and 7.

### Impact on glutamate receptors

Glutamate receptors have also been shown to be involved in GC-mediated effects in a context of neuropathic pain. In this framework, the role of the metabotropic receptor mGluR5 has been investigated under conditions of GR agonist and antagonist treatment. Kalariti and colleagues [44] demonstrated *in vitro* that dexamethasone administration increased mGluR5 mRNA and protein expression. This result is consistent with our observation at day 4, where a large increase of mGluR5 mRNA expression was seen in the DEX group. However, these differences had vanished at day 21, when the groups also displayed similar mechanical pain thresholds. These findings seem to support the assumption that mGluR5 could be more implicated in the onset rather than in the maintenance of the hypersensitivity observed in the DEX group.

Glucocorticoid-mediated effects on NMDA receptor regulation have also been studied quite extensively. It has e.g. been shown that GR activation leads to an upregulation of the NMDA receptor expression in the hippocampus and that enhanced GCs levels lead to an increase in glutamatergic transmission [21]. In addition, after a peripheral nerve injury, a time-dependent and region-specific upregulation of two subunits of the NMDA receptor has been observed [11]. More precisely, NR1 and NR2 mRNA expression have been shown to be increased in the ipsilateral spinal dorsal horn in a CCI model. Furthermore, these subunit expressions decrease under conditions of RU-486 administration. These data are in agreement with our results showing a dexamethasone-related enhancement of mRNA expression of NR1 four days after surgery in the ipsilateral spinal cord. This increased expression is accompanied by pain hypersensitivity exhibited by the DEX group rats at this early time point. Moreover, we can confirm an antagonist-related downregulation of the NR2 subunit in later phases of experimentally induced CCI.

In our hands the NMDA receptor subunit NR1 played a role in the onset rather than in the maintenance of mechanical allodynia/hyperalgesia whereas the NR2a subunit could subsequently have taken over a more dominant role.

### Limitations of the study

A limitation of the study is that we only present mRNA expression data for the spinal mediators. Regulations on a post-transcriptional level, e.g. via micro-RNAs could play an important role. Therefore, measurement of protein expression via western blotting should be included in further experiments. It should however be noted that the reliability of this kind of protein quantification (by using house keeping genes) is currently also under debate.

### Conclusion

The present study demonstrated that at an early time point post-CCI, the activation of GR leads to an enhancement of mRNA expression of glutamate receptors accompanied by an increase in mechanical pain sensitivity.

Under these conditions, GR activation may hence lead to an intensification of glutamatergic transmission that may outweigh putative anti-inflammatory effects. Further investigations aimed at confirming this assumption and at elucidating the underlying mechanisms will have to include the assessment of protein levels. The involvement of altered descending pain control pathways will also have to be considered.

## References

[1] Davis MC, Zautra AJ, Reich JW (2001) Vulnerability to stress among women in chronic pain from fibromyalgia and osteoarthritis. Ann Behav Med 23: 215–26. 11495222 10.1207/S15324796ABM2303_9

[2] Aloisi AM, Buonocore M, Merlo L, Galandra C, Sotgiu A, et al (2011) Chronic pain therapy and hypothalamic-pituitary-adrenal axis impairment. Psychoneuroendocrino 36: 1032–1039. 10.1016/j.psyneuen.2010.12.017 21256679

[3] Kanczkowski W, Alexaki VI, Tran N, Großklaus S, Zacharowski K, et al (2013) Hypothalamo-pituitary and immune-dependent adrenal regulation during systemic inflammation. P Natl Acad Sci USA 110: 14801–14806. 10.1073/pnas.1313945110 PMC3767499 23959899

[4] Straub RH, Bijlsma JW, Masi A, Cutolo M (2013) Role of neuroendocrine and neuroimmune mechanisms in chronic inflammatory rheumatic diseases -The 10-year update. Semin Arthritis Rheum in press. doi:10.1016/j.semarthrit.2013.04.008. 10.1016/j.semarthrit.2013.04.008 23731531

[5] Kuehl LK, Michaux GP, Richter S, Schächinger H, Anton F (2010) Increased basal mechanical pain sensitivity but decreased perceptual wind-up in a human model of relative hypocortisolism. Pain 149: 539–46. 20381248 10.1016/j.pain.2010.03.026

[6] Michaux GP, Magerl W, Anton F, Treede RD (2012) Experimental characterization of the effects of acute stresslike doses of hydrocortisone in human neurogenic hyperalgesia models. Pain 153: 420–428. 22178393 10.1016/j.pain.2011.10.043

[7] Chrousos GP (2000) Stress, chronic inflammation, and emotional and physical well-being: Concurrent effects and chronic sequelae. J Allergy Clin Immunol 106: S275–291. 11080744 10.1067/mai.2000.110163

[8] Newton R (2000) Molecular mechanisms of glucocorticoid action: what is important? Thorax 55: 603–613. 10856322 10.1136/thorax.55.7.603 PMC1745805

[9] Lee SW, Tsou AP, Chan H, Thomas J, Petrie K, et al (1988) Glucocorticoids selectively inhibit the transcription of the interleukin 1 beta gene and decrease the stability of interleukin 1 beta mRNA. Proc Natl Acad Sci USA 85: 1204–1208. 3257575 10.1073/pnas.85.4.1204 PMC279735

[10] Liberman AC, Druker J, Perone MJ, Arzt E (2007) Glucocorticoids in the regulation of transcription factors that control cytokine synthesis. Cytokine Growth F R 18: 45–56. 10.1016/j.cytogfr.2007.01.005 17336577

[11] Wang S, Lim G, Zeng Q, Sung B, Yang L, et al (2005) Central glucocorticoid receptors modulate the expression and function of spinal NMDA receptors after peripheral nerve injury. J Neurosci 25: 488–495. 15647493 10.1523/JNEUROSCI.4127-04.2005 PMC6725479

[12] Takasaki I, Kurihara T, Saegusa H, Zong S, Tanabe T (2005) Effects of glucocorticoid receptor antagonists on allodynia and hyperalgesia in mouse model of neuropathic pain. Eur J Pharmacol 524: 80–83. 16256102 10.1016/j.ejphar.2005.09.045

[13] Bennett GJ, Xie YK (1988) A peripheral mononeuropathy in rat that produces disorders of pain sensation like those seen in man. Pain 33: 87–107. 2837713 10.1016/0304-3959(88)90209-6

[14] Gómez-Nicola D, Valle-Argos B, Suardíaz M, Taylor JS, Nieto-Sampedro M (2008) Role of IL-15 in spinal cord and sciatic nerve after chronic constriction injury: regulation of macrophage and T-cell inflitration. J Neurochem 107: 1741–1752. 19014377 10.1111/j.1471-4159.2008.05746.x

[15] Oprée A, Kress M (2000) Involvement of the proinflammatory cytokines tumor necrosis factor-alpha, IL-1 beta, and IL-6 but not IL-8 in the development of heat hyperalgesia: effects on heat-evoked calcitonin gene-related peptide release from rat skin. J Neurosci 20: 6289–6293. 10934280 10.1523/JNEUROSCI.20-16-06289.2000 PMC6772609

[16] Junger H, Sorkin LS (2000) Nociceptive and inflammatory effects of subcutaneous TNFalpha. Pain 85: 145–151. 10692613 10.1016/s0304-3959(99)00262-6

[17] Milligan ED, Watkins LR (2009) Pathological and protective roles of glia in chronic pain. Nat Rev Neurosci 10: 23–36. 19096368 10.1038/nrn2533 PMC2752436

[18] Coutinho AE, Chapman KE (2011) The anti-inflammatory and immunosuppressive effects of glucocorticoids, recent developments and mechanistic insights. Mol Cell Endocrinol 335: 2–13. 20398732 10.1016/j.mce.2010.04.005 PMC3047790

[19] Alexander JK, DeVries AC, Kigerl KA, Dahlman JM, Popovich PG (2009) Stress exacerbates neuropathic pain via glucocorticoid and NMDA receptor activation. Brain Behav Immun 23: 851–860. 19361551 10.1016/j.bbi.2009.04.001 PMC2735409

[20] Wang S, Lim G, Zeng Q, Sung B, Ai Y, et al (2004) Expression of central glucocorticoid receptors after peripheral nerve injury contributes to neuropathic pain behaviors in rats. J Neurosci 24: 8595–8605. 15456833 10.1523/JNEUROSCI.3058-04.2004 PMC6729915

[21] Lu J, Goula D, Sousa N, Almeida OF (2003) Ionotropic and metabotropic glutamate receptor mediation of glucocorticoid-induced apoptosis in hippocampal cells and the neuroprotective role of synaptic N-methyl-d-aspartate receptors. Neuroscience 121: 123–131. 12946705 10.1016/s0306-4522(03)00421-4

[22] Urban MO, Hama AT, Bradbury M, Anderson J, Varney MA, et al (2003) Role of metabotropic glutamate receptor subtype 5 (mGluR5) in the maintenance of cold hypersensitivity following a peripheral mononeuropathy in the rat. Neuropharmacology 44: 983–993. 12763091 10.1016/s0028-3908(03)00118-7

[23] Chaplan SR, Bach FW, Pogrel JW, Chung JM, Yaksh TL (1994) Quantitative assessment of tactile allodynia in the rat paw. J Neurosci Methods 53: 55–63. 7990513 10.1016/0165-0270(94)90144-9

[24] Pfaffl M (2001) A new mathematical model for relative quantification in real- time RT-PCR. Nucleic Acids Res 29: 2002–2007. 10.1093/nar/29.9.e45 PMC55695 11328886

[25] Soro A, Panarelli M, Holloway CD, Fraser R, Keuron CJ (1995) Effects of the glucocorticoid antagonist RU 486 in spontaneously hypertensive and Sprague Dawley rats. J Endocrinol Invest 18: 833–839. 8778154 10.1007/BF03349829

[26] Ou GK, Wang RX, Li JJ, Cao H, Lian QQ, et al (2013) Effect of M8046 on expression of COX-2/PGE2 in spinal cord and DRG in rats with neuropathic pain. Zhongguo Zhong Yao Za Zhi 29: 97–100. 23833955

[27] Osikowicz M, Mika J, Przewlocka B (2013) The glutamatergic system as a target for neuropathic pain relief. Exp Physiol 98: 372–384. 23002244 10.1113/expphysiol.2012.069922

[28] McEwen BS, Kalia M (2010) The role of corticosteroids and stress in chronic pain conditions. Metabolism 59 (Suppl 1)S9–S15. 20837196 10.1016/j.metabol.2010.07.012

[29] Kingery WS, Agashe GS, Sawamura S, Davies F, Clark JD, et al (2001) Glucocorticoid inhibition of neuropathic hyperalgesia and spinal Fos expression. Anesth Analg 92: 476–482. 11159254 10.1097/00000539-200102000-00037

[30] Milligan ED, Twining C, Chacur M, Biedenkapp J, O'Connor K, et al (2003) Spinal glia and proinflammatory cytokines mediate mirror-image neuropathic pain in rats. J Neurosci 23: 1026–1040. 12574433 10.1523/JNEUROSCI.23-03-01026.2003 PMC6741915

[31] Wen YR, Suter MR, Kawasaki Y, Huang J, Pertin M, et al (2007) Nerve conduction blockade in the sciatic nerve prevents but does not reverse the activation of p38 mitogen-activated protein kinase in spinal microglia in the rat spared nerve injury model. Anesthesiology 107: 312–321. 17667577 10.1097/01.anes.0000270759.11086.e7

[32] Li H, Xie W, Strong JA, Zhang J-M (2007) Systemic anti-inflammatory corticosteroid reduces mechanical pain behaviour, sympathetic sprouting, and elevation of pro-inflammatory cytokines in a rat model of neuropathic pain. Anesthesiology 107: 489–477. 10.1097/01.anes.0000278907.37774.8d PMC2174791 17721250

[33] Leung L, Cahill C (2010) TNF-α and neuropathic pain - a review. J Neuroinflamm 7: 27–39. 10.1186/1742-2094-7-27 PMC2861665 20398373

[34] McMahon SB, Cafferty WB, Marchand F (2005) Immune and glial cell factors as pain mediators and modulators. Exp Neurol 192: 444–462. 15755561 10.1016/j.expneurol.2004.11.001

[35] Snyder DS, Unanue ER (1982) Corticosteroids inhibit murine macrophage Ia expression and interleukin 1 production. J Immunol 129: 1803–1805. 6811653

[36] Clark A (2000) Post-transcriptional regulation of pro-inflammatory gene expression. Arthritis Res 2: 172–174. 11094425 10.1186/ar83 PMC129998

[37] Ji R-R, Berta T, Nedergaard M (2013) Glia and pain: is chronic pain a gliopathy? Pain 154 (Suppl 1)S10–S28. 23792284 10.1016/j.pain.2013.06.022 PMC3858488

[38] Abraham SM, Lawrence T, Kleiman A, Warden P, Medghalchi M, et al (2006) Antiinflammatory effects of dexamethasone are partly dependent on induction of dual specificity phosphatase 1. J Exp Med 203: 1883–1889. 16880258 10.1084/jem.20060336 PMC2118371

[39] Dawson J, Rordorf-Adam C, Geiger T, Towbin H, Kunz S, et al (1993) Interleukin-1 (IL-1) production in a mouse tissue chamber model of inflammation. II. Identification of (tissue) macrophages as the IL-1 producing cells and the effect of anti-inflammatory drugs. Agents Actions 38: 255–264. 8213352 10.1007/BF01976218

[40] Swantek JL, Cobb MH, Geppert TD (1997) Jun N-terminal kinase/stress-activated protein kinase (JNK/SAPK) is required for lipopolysaccharide stimulation of tumor necrosis factor alpha (TNF-alpha) translation: glucocorticoids inhibit TNF-alpha translation by blocking JNK/SAPK. Mol Cell Biol 17: 6274–6282. 9343388 10.1128/mcb.17.11.6274 PMC232478

[41] Yan P, Liu N, Kim GM, Xu J, Li Q, et al (2003) Expression of the type 1 and type 2 receptors for tumor necrosis factor after traumatic spinal cord injury in adult rats. Exp Neurol 183: 286–297. 14552870 10.1016/s0014-4886(03)00135-3

[42] Aschner M (1998) Astrocytes as mediators of immune and inflammatory responses in the CNS. Neurotoxicology 19: 269–281. 9553964

[43] Wen ZH, Wu GJ, Chang YC, Wang JJ, Wong CS (2005) Dexamethasone modulates the development of morphine tolerance and expression of glutamate transporters in rats. Neuroscience 133: 807–817. 15893883 10.1016/j.neuroscience.2005.03.015

[44] Kalariti N, Lembessis P, Papageorgiou E, Pissimissis N, Koutsilieris M (2007) Regulation of the mGluR5, EAAT1 and GS expression by glucocorticoids in MG-63 osteoblast-like osteosarcoma cells. J Musculoskelet Neuronal Interact 7: 113–118. 17627080

